# *Daemonorops draco* Blume Induces Apoptosis Against Acute Myeloid Leukemia Cells *via* Regulation of the miR-216b/c-Jun

**DOI:** 10.3389/fonc.2022.808174

**Published:** 2022-03-09

**Authors:** Moon Nyeo Park, Hee Won Jeon, Md. Ataur Rahman, Se Sun Park, Se Yun Jeong, Ki Hyun Kim, Sung-Hoon Kim, Woojin Kim, Bonglee Kim

**Affiliations:** ^1^ Department of Pathology, College of Korean Medicine, Kyung Hee University, Seoul, South Korea; ^2^ Korean Medicine-Based Drug Repositioning Cancer Research Center, College of Korean Medicine, Kyung Hee University, Seoul, South Korea; ^3^ School of Pharmacy, Sungkyunkwan University, Suwon, South Korea

**Keywords:** *Daemonorops draco* Blume, acute myeloid leukemia, apoptosis, miR-216b, c-Jun, ER stress, reactive oxygen species

## Abstract

*Daemonorops draco* Blume (DD), also called dragon’s blood, has been used as a traditional Korean medicine, especially for relieving pain caused by wound infection. Recently, it has been described that DD has antibacterial and analgesic effects. In this study, the underlying anticancer effect of DD associated with apoptosis was investigated in acute myeloid leukemia cell lines U937 and THP-1. DD exhibited cytotoxic effects and induced apoptosis in U937 and THP-1 cells. Moreover, DD treatment significantly reduced mitochondrial membrane potential (ΔΨ). The protein expression of cleaved poly(ADP-ribose) polymerase, cleaved caspase-3, p-H2A.X, CCAAT/enhancer-binding protein (CHOP), and activating transcription factor 4 was upregulated by DD treatment. Consistently, DD-treated cells had increased reactive oxygen species (ROS) level in a concentration-dependent manner *via* miR-216b activation in association with c-Jun inhibition. N-acetyl-L-cysteine pretreatment reversed the cytotoxic effect of DD treatment as well as prevented ROS accumulation. Collectively, the results of this study suggest that the anticancer effect of DD in AML was mediated by CHOP-dependent apoptosis along with ROS accumulation and included upregulation of miR-216b followed by a decrease in c-Jun.

## Introduction

Acute myeloid leukemia (AML) is a heterogeneous malignant disease caused by uncontrolled proliferation of immature myeloid blast cells. The expansion of myeloid precursor cells in the bone marrow (BM) is a distinct pathological characteristic of AML, which disrupts hematopoiesis in the BM (1). More than half of patients with AML have chromosomal abnormalities, while the remaining 40%–50% had cytogenetically normal AML (CN-AML). Various genetic mutations or changes in gene phenotypes are detected in patients with CN-AML, which are important in determining prognosis and treatment (2). To date, 13 types of mutant genes have been discovered, including *Nucleophosmin 1 (NPM1)*, *DNA methyltransferase 3A (DNMT3A)*, *FMS-like tyrosine kinase 3 (FLT3), Isocitrate dehydrogenase (IDH)*, and *Ten–eleven-translocation 2 (TET2)* (3). These chromosomal and gene mutations were used as an index for the four stages of risk stratification in the 2010 European Leukemia Net classification scheme (4).

The pathology and prognosis of AML are closely related to endoplasmic reticulum (ER) stress and the amount of reactive oxygen species (ROS). Doron et al. have reported that AML cells utilize ER stress to change the stromal composition in the BM. Therefore, the reduction of ER stress, change in ROS, and correlation between the two are important indicators for evaluating AML activity (5). Moreover, ROS was related to the lifespan of hematopoietic stem cells and alterations in leukemic oncogenes (6). The ER maintains cellular functions, including the synthesis and proper folding of proteins (7, 8). However, in undesirable conditions, such as hypoxia, ischemia, and turbulence in intracellular pH, ER stress occurs (9). Subsequently, unfolded protein response is induced by ER stress, which results in various symptoms, including neurodegenerative diseases and cancer (10). Thus, ER stress is one of the key mechanisms in the process of ROS-mediated apoptosis (11).

MicroRNAs (miRNA) play an essential role in maintaining homeostasis of cellular growth, differentiation, migration, and apoptosis, which are regulated by the development and differentiation of hematopoietic cells (12). In particular, alterations in miRNA genes have close relationships with the development of tumor and hematological diseases (13, 14). Impaired miRNA expression in AML that can stem from many causes, such as chromosome translocations, inversion, gene deletions, and mutations, is directly or indirectly controlled by post-transcriptional modification due to limitless clonal expansion of myeloid blast cells (15). Amanda et al. have reported that 33 types of miRNA were upregulated or downregulated in AML, suggesting the use of miRNAs in subclassifying the types of leukemia (16). Moreover, miR-15 and miR-16 were deleted or downregulated in chronic lymphocytic leukemia (17), whereas overexpression of the miR-181 family is associated with the high risk of cytogenetically normal AML along with CCAAT/enhancer-binding protein-alpha mutations (18). Modulation of miRNA genes is related to the mutation of different oncogenes, as miR-155 was regulated in patients with AML with *FLT3*-internal tandem duplication mutations, and miR-10a and miR-10b were capable predictors of AML with mutations (19, 20). Notably, miR-216b is downregulated in various types of cancer, including cervical cancer, non-small cell lung cancer (NSCLC), and colorectal cancer (21–23). The expression of miR-216b showed a higher frequency of *U2AF1* and *IDH1/2* mutations in patients with AML and was a valuable predictor of AML recurrence (24).

The proto-oncoprotein c-Jun is an initial transcription factor that regulates the expression of cellular mechanisms and carcinogen combination, which belongs to the Activation protein-1 (AP-1) family (25). The overexpression of c-Jun is superior to the mechanism caused by ER stress-related apoptosis, suppressing the death caused by the activation of cleaved caspase-3 and cleaved poly(ADP-ribose) polymerase (PARP) (26). Several studies have identified c-Jun as a target protein of miR-216b, which was effective in alleviating cancer-related symptoms. Xu et al. confirmed that miR-216b directly targeted c-Jun, consequently inhibiting AP-1-dependent transcription, and was susceptible to ER stress-related apoptosis (26). Overexpression of miR-216b improved cisplatin-induced apoptosis in NSCLC, which was mediated by inhibiting the expression of c-Jun (27). Hence, changes in c-Jun activity through the regulation of miR-216b will be a standard for observing changes in AML cell activity.

*Daemonorops draco* Blume (DD), a traditional medicine derived from a natural resin, is widely used for its analgesic effects in wound healing, ulcers, and diarrhea and has also hemostatic, anti-inflammatory effects and reduces genesis of osteoclasts (28, 29). DD is also referred to as dragon’s blood; however, this name collectively refers to plant extracts of various origins according to region (6). Although other types of dragon’s blood have shown antitumor effects, such as inhibiting liver cancer (30, 31), the effects of DD in treating cancer have yet to be explored. Flavone compounds derived from DD form a phenolic group, which has antioxidant and anti-inflammatory activities and properties that alleviate cancer toxicity (32, 33). Therefore, to determine various bioactive components derived from DD, liquid chromatography (LC)/mass spectrometry (MS)/ultraviolet detection (UV) was performed. In this study, the anticancer effect of DD was investigated; moreover, this study evaluated the relationship between DD and ER stress and ROS and attempted to verify the detailed mechanisms at a molecular level.

## Materials and Methods

### Materials

DD was cultivated in Kang Won province in Korea and was bought at Yak Won Herbal Pharmacy. DD was stored at the herbarium of the Department of Pathology, College of Korean Medicine, and Kyung Hee University. DD (200 g) was extracted using 99% ethyl alcohol (Duksan, Gyeonggi-do, South Korea) according to the procedure described in previous studies (34). Briefly, the solution was concentrated to 100-ml aqueous solution using an evaporator and kept at −80°C for 24 h. Then, DD was dissolved in dimethyl sulfoxide (Duksan, Gyeonggi-do, South Korea). DD stock was prepared to a concentration of 200 mg/ml and then stored at −20°C.

### LC/MS/UV-Based Analysis for DD Extract

The extract of DD was prepared by dissolving the samples in methanol. The solutions were filtered through a 0.45-mm hydrophobic polytetrafluoroethylene filter and analyzed by LC/MS using an Agilent 1200 Series HPLC system (Agilent Technologies, Santa Clara, CA) equipped with a photodiode array detector combined with a 6130 Series electrospray ionization (ESI) mass spectrometer. The ESI conditions were set as follows: capillary voltage, 2.0 kV; convoltage, 50 V; source temperature, 120°C, desolvation temperature, 350°C; and desolvation gas flow rate, 800 L/h. High-purity nitrogen was used as the nebulizer and auxiliary gas. The collision energy for detecting the precursor ions was set to 3 eV. Analysis was performed by injecting 5 μl of the sample using Aglient Eclipse Plus C_18_ column (3.5 μm, 4.6 mm × 100 mm) set at 35°C. The mobile phase consisting of formic acid in H_2_O (0.1% [v/v]) (A) and methanol (B) was delivered at a flow rate of 0.3 ml/min by applying the following programmed gradient elution: 0%–100% (B) for 30 min, 100% (B) for 1 min, 100% (B) isocratic for 10 min, and then 0% (B) isocratic for 10 min, to perform post-run reconditioning of the column.

### Cell Culture

The AML cell lines THP-1 and U937 were purchased from Korean Cell Line Bank (Seoul, South Korea). THP-1 and U937 were cultured in RPMI 1640 medium containing 10% fetal bovine serum, 10,000-U/ml penicillin/streptomycin, and 2-μM L-glutamine (Gibco, Grand Island, NY, USA). All cells were cultured in an incubator at 37°C in a humidified incubator containing 5% CO_2_.

### Cytotoxicity Assay

A cytotoxicity assay was performed to examine THP-1 and U937 cells using EZ-Cytox Cell Viability Assay Kit (Daeil Lab Service, Seoul, South Korea) according to the manual. Cells were seeded and exposed to various concentrations of DD (i.e., 12.5, 25, 50, 100, and 200 μg/ml) for 24 h onto a 96-well plate. The cells were incubated with an EZ-Cytox solution until formazan was formed for 2 h. The absorbance values were measured at 450 nm using a microplate reader (Bio-Rad, Hercules, CA, USA).

### Mitochondrial Membrane Potential Assay

JC1-MMP Assay Kit (ab113850, Abcam) was used. JC-1 Dye (Mitochondria Function Assay Kit, Thermo–Fisher Scientific, USA) for MMP can be detected using aggregated (excitation/emission = 535/595) and J-monomers (excitation/emission = 475/535). The signal ratio can be used to differentiate healthy mitochondria from depolarized ones in association with changes in mitochondrial calcium, superoxide, mitochondrial permeability transition, and membrane potential. THP-1 and U937 cells were seeded in a 96-well plate and pretreated with a density of 1 × 10^6^ cells per well. After staining with 20-μM JC-1 Dye for 10 min at room temperature (RT) in the dark, the cells were treated with DD (15 and 30 μg/ml) for 4 h. Then, the 96-well plates were measured using an enzyme-linked immunosorbent assay (ELISA) reader (Bio-Rad, Hercules, CA, USA).

### Western Blotting

Cells were lysed with a lysis buffer (pH = 7.4, 150-mM NaCl, 1% NP-40, 50-mM Tris-HCl, 0.25% sodium deoxycholic acid, 1-M ethylenediaminetetraacetic acid, 1-mM Na_3_VO_4_, and 1-mM NaF) containing a protease inhibitor cocktail (Amresco, Scolon, OH, USA). In the lysate sample, the protein concentration was quantified using Bio-Rad DC Protein Assay Kit II (Bio-Rad, Hercules, CA, USA) according to the manufacturer’s instructions. The proteins were separated using sodium dodecyl sulfate–polyacrylamide gel electrophoresis (8%–12%) by electrophoresis and transferred to polyvinylidene fluoride membranes (Millipore, USA). Then, 5% skim milk in Tris-buffered saline plus 0.1% Tween 20 (TBST) was used to block nonspecific protein binding sites. The following specific primary antibodies were used—c-PARP (1:1,000) (#9542) (Cell Signaling, Beverly, MA, USA), c-cas3 (1:1,000) (#9661), CCAAT/enhancer-binding protein (CHOP) (1:1,000) (#2895), p-H2A.X (1:1,000) (#2577), β-actin (1:1,000) (# 4967), p-ATF4 (1:1,000) (#PA5-105835) (Thermo–Fisher Scientific, Waltham, MA, USA), and p-c-Jun (1:1,000) (#822) (Santa Cruz Biotechnologies, Santa Cruz, CA, USA)—for 24 h at 4°C. After washing with TBST for 30 min, the membranes were incubated with rabbit horseradish peroxidase-conjugated immunoglobulin G (IgG) secondary anti-mouse or rabbit antibody (5% skim milk) (1:10,000, Santa Crus, Dallas, TX, USA) for 1 h at RT. Protein expression levels were identified using an enhanced chemiluminescence system (Amersham Pharmacia, Piscataway, NJ, USA).

### Live and Dead Cell Assays

THP-1 (2 × 10^5^ cells/ml) or U937 (2 × 10^5^ cells/ml) cells were seeded into a 4-chamber slide (Nunc™ Lab-Tek™ II Chamber Slide™ System, Thermo–Fisher Scientific, USA) at 1 ml/well. After seeding, the culture medium was treated with 30-μg/ml DD for 24 h at 1 ml/well. The cells were washed with Dulbecco’s phosphate-buffered saline, then loaded with calcein-AM (LIVE/DEAD^®^ Viability/Cytotoxicity Kit, Thermo–Fisher Scientific, USA) and ethidium homodimer-1 (LIVE/DEAD^®^ Viability/Cytotoxicity Kit, Thermo–Fisher Scientific, USA) for 30 min, and added to each slide, according to the manufacturer’s protocol. Images were obtained using confocal microscopy FV10i (OLYMPUS Fluoview USA) (green: live cells; red: dead cells; scale bar = 100 μm).

### Measurement of ROS

The Reactive Oxygen Species Detection Assay (Abcam, Cambridge, United Kingdom) using reagent 2’,7’-dichlorofluorescin diacetate (DCFDA) was used to identify hydroxyl, peroxyl, and other ROS of cellular cytosolic hydrogen peroxide (H_2_O_2_). THP-1 and U937 cells were seeded onto 96-well plates and pretreated with N-Acetyl-L-cysteine (NAC) (Sigma Aldrich Co., St. Louis, MO, USA) for 1 h, and the control group was not pretreated with NAC. Then, the cells were stained with 20-µM DCFDA for 30 min at RT in the dark. Consequently, both THP-1 and U937 cells were treated with 30-μg/ml DD for 4 h. Then, the 96-well plates were measured using an ELISA reader (Bio-Rad, Hercules, CA, USA) (Ex/Em = 450/570 nm)

### Quantitative Real-Time Polymerase Chain Reaction

Total RNA was isolated using the RNeasy Mini Kit (EZ™ Total RNA Mini Prep Kit, Enzynomics, South Korea) according to the manufacturer’s protocol and reverse transcribed using the HB_I RT Reaction Kit. cDNAs were amplified by qRT-PCR using the synthesized specific HB_I Nucleic Mix II primers and RNU6B HB primers (HeimBiotek, South Korea). PCR was performed using the LightCycler instrument (Roche Applied Sciences, Indianapolis, IN, USA). PCR was started at 95°C for 15 min, followed by 40 cycles at 95°C for 10 s and 60°C for 40 s, and finished with 95°C for 60 s, 55°C for 30 s and 95°C for 30 s. The expression of RNU6B was used to normalize the expression of target genes. The specific primer Has-miR-216b was designed and synthesized by HeimBiotek Company (HeimBiotek, South Korea). Relative miRNA fold change was normalized using standard C_t_ values of RNU6B (U6) (HeimBiotek, South Korea). RT-PCR was performed using the LightCycler instrument (Roche Applied Science, Indianapolis, IN, USA).

### Transfection miR-216b Inhibitor Study

THP-1 and U937 cells were transfected with a miR-216b inhibitor (HeimBiotek, South Korea) and ViaFect™ Transfection Reagent (Promega, Madison USA) and seeded onto 6-well plates with prewarmed serum-free medium. In this process, 10–50-nM miR-216b inhibitor and 3-μl ViaFect™ Transfection Reagent were added into 100-μl prewarmed serum-free medium at RT and mixed immediately. The cells were incubated with ViaFect™ Transfection Reagent: miR-216b inhibitor mixtures for 5 min transfected using ViaFect™ Transfection Reagent according to the manufacturer’s protocol. After the transfection of miR-216b inhibitor for 48 h, THP-1 and U937 cells were treated with 30-μg/ml DD for 24 h. MiR-216b inhibitor oligobase type with follow: 2’ O-Methyl RNA base was applied by HeimBiotek, South Korea.

### Statistical Analysis

Data were presented as means ± standard deviation. Statistically significant differences between the control and MLT-treated groups were calculated using Student’s *t*-test using SigmaPlot 12 (SysTest Software Inc., San Jose, CA, USA). All experiments were performed in triplicate. Differences with *p*-values of less than 0.05 were considered statistically significant.

## Results

### Identification of Various Flavonoids in DD by HPLC-MS

**Figure 1:**
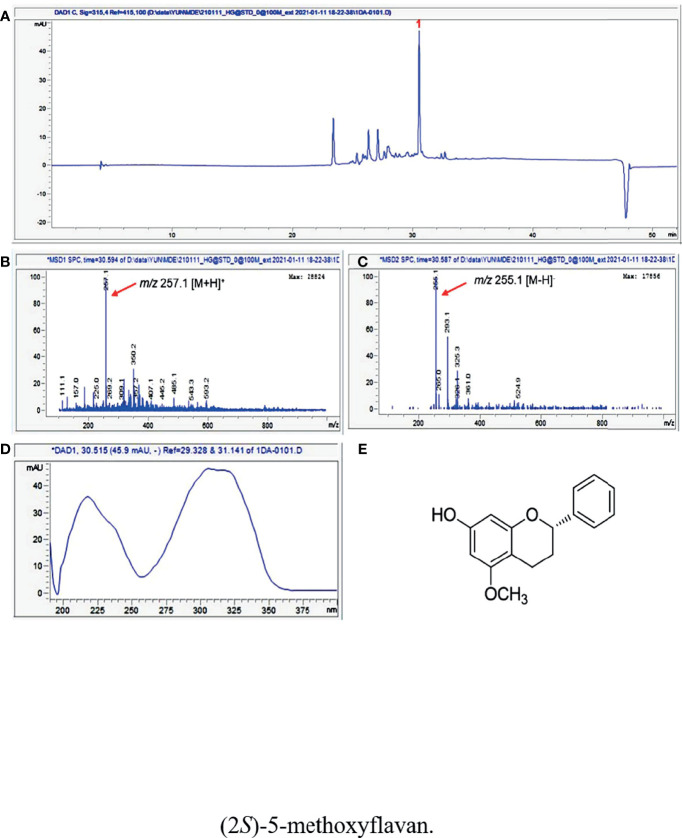
**(A)** UV chromatogram of LC/MS (detection wavelength was set at 315 nm) of the extract of DD. **(B)** Positive and **(C)** negative ion-mode ESI-MS data of the peak at retention time 30.5 min and **(D)** UV data of the peak. **(E)** The chemical structure of (2*S*)-7-hydroxy-5-methoxyflavan (1).

LC/MS/UV-based analysis of the extract of DD revealed a major peak with molecular ions of *m/z* 257.1 [M+H]^+^ and *m/z* 255.1 [M-H]^-^ at a retention time of 30.5 min, which also showed a unique UV spectrum (λ_max_ 218, 236, 304, and 321 nm) (**Figure 1**). Based on the characteristic UV data and the molecular ions detected by LC/MS, as well as the chemical database of DD previously reported in available studies (29, 34–36), the major metabolite was determined to be (2*S*)-7-hydroxy-5-methoxyflavan (**Figure 1**).

### DD Had a Cytotoxic Effect on AML Cells

**Figure 2:**
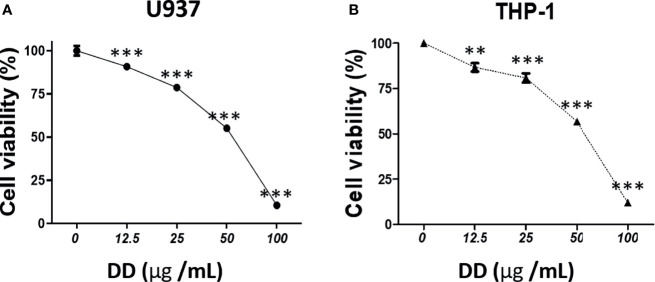
*Daemonorops draco* Blume (DD) exerted a cytotoxic effect on acute myeloid leukemia cells. The cytotoxicity of DD in **(A)** U937 and **(B)** THP-1 cells. The cells were treated with DD (i.e., 12.5, 25, 50, 100, or 200 μg/ml) for 24 h. Cell viability assay was performed using EZ-Cytox. Values above represent the means of three experiments. Means ± standard deviation; **p < 0.01 and ***p < 0.001 compared with the untreated groups.

To investigate the cytotoxic effect of DD, EZ-Cytox was performed in AML cells, including U937 and THP-1 ells. In **Figures 2A, B**, the concentrations of 25 and 50 μg/ml showed a survival rate of approximately 55%–80%. Cell viability assay showed that the survival rate decreased in a concentration-dependent manner. This result was statistically significant.

### DD Reduced MMP and Induced Apoptosis in AML Cells

**Figure 3:**
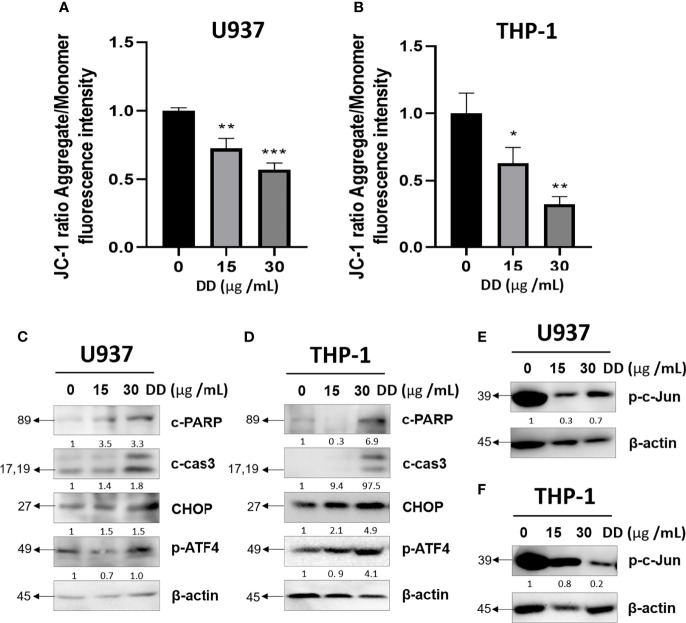
*Daemonorops draco* Blume (DD) reduced mitochondrial membrane potential and induced apoptosis in U937 and THP-1 cells treated with DD. **(A, B)** Cells were pretreated at JC-1 (20 μM) and DD (15 and 30 μg/ml) for 4 h. Green monomeric fluorescence form changed to red fluorescent aggregates in a concentration-dependent manner, which was measured using a microplate reader. (C–F) After treatment with DD (15 and 30 μg/ml) for 24 h, the cells were subjected to Western blotting due to the expression of apoptosis-related proteins, such as cleaved caspase-3, cleaved poly(ADP-ribose)polymerase, CCAAT/enhancer-binding protein, p-ATF4, p-c-Jun, and β-actin, in **(C, E)** U937 and **(D, F)** THP-1 cells. Fluorescein isothiocyanate (excitation/emission = 540/570) and rhodamine (excitation/emission = 540/570). Values above represent the means of three experiments. Means ± standard deviation; *p < 0.05, **p < 0.01, and ***p < 0.001 compared with the untreated groups.

To establish the mechanism of apoptosis controlled by DD, JC-1 staining and Western blotting were conducted. As shown in **Figures 3A, B**, DD reduced MMP (ΔΨ) in a concentration-dependent manner in AML cells. Furthermore, caspase-3 is a critical executioner of mitochondria-mediated apoptosis (37). Western blotting showed that DD significantly induced the activation of caspase-3 along with the expression of cleaved PARP *via* the inhibitory regulation of c-Jun. Furthermore, CHOP is a major mediator of ER stress-related pathways closely related to caspase-3 activation (38, 39). CHOP alone does not exert sufficient effect to cause cell destruction but enhances the effect of activating transcription factor 4 (ATF4) to decrease cell viability through ER stress. Furthermore, ATF4 and CHOP were found to act on the same target gene to increase protein synthesis related to stress-induced transcription, inducing apoptosis (40). Nevertheless, the underlying anticancer mechanism of DD related to ER stress-related apoptotic proteins and caspase-mediated apoptosis has not been identified so far. Furthermore, DD significantly reduced MMP (ΔΨ), cleaved caspase 3, and cleaved PARP; increased CHOP and p-ATF4; and attenuated p-c-Jun in a concentration-dependent manner in AML cells (**Figures 3C–F**). These results demonstrated that DD is involved in apoptosis *via* the mitochondria-mediated caspase and ER stress-related apoptosis activation pathways.

### DD Increased DNA Damage in AML Cells

**Figure 4:**
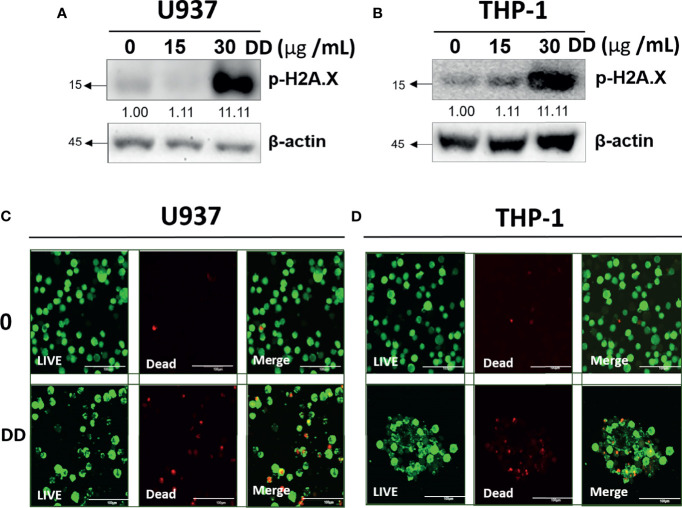
*Daemonorops draco* Blume (DD) increased DNA damage in U937 and THP-1 cells. **(A, B)** The effect of DD on H2A.X, which was treated with various concentrations of DD (i.e., 15 and 30 μg/ml for 24 h), in U937 and THP-1 cells, which were subjected to Western blotting with the antibodies of p-H2A.X and β-actin. **(C, D)** Confocal images of AML cells represented live cells (left panel), dead cells (middle panel), and the combination of both (right panel). AML cells were treated with DD (30 μg/ml) for 24 h, and double dyes were incubated at 37°C for 30 min. AML cells were stained with calcium AM (excitation/emission = 494/517) and ethidium homodimer-1 (excitation/emission = 528/617). Scale bar = 100 μm.

To evaluate the cytotoxic effects of DD, Western blotting and live/dead staining were adopted in U937 and THP-1 cells. DD significantly increased p-H2A.X by Western blotting in a concentration-dependent manner compared with the untreated groups (**Figures 4A, B**). Similarly, DD-treated cells emitted significantly more red fluorescence due to dead cells compared with the control group (**Figures 4C, D**). Consistently, DD effectively induced apoptosis by causing DNA damage.

### DD Increased ROS and NAC Reversed DD-Induced Cytotoxicity in AML Cells

**Figure 5:**
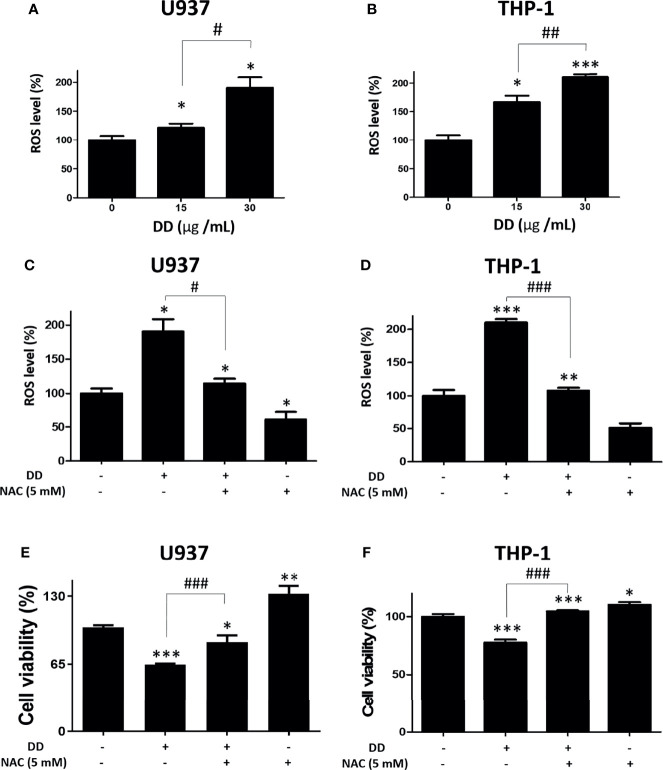
*Daemonorops draco* Blume (DD) increased reactive oxygen species (ROS) accumulation and N-Acetyl-L-cysteine (NAC) pretreatment reversed the cytotoxic effect of DD in U937 and THP-1 cells. **(A, B)** Both cells were incubated with 20-μM 2’,7’-dichlorofluorescin diacetate (DCFDA) for 30 min at 37°C in the dark and subjected to ROS assay. The cells were exposed to 30-μg/ml DD for 4 h. DCFDA fluorescence was determined using a dual microplate reader. **(C, D)** Both cells were exposed to NAC (5 mM) pretreatment for 60 min and subjected to ROS measurement. **(E, F)** A cell viability assay was conducted with absorbance measurement using an optical spectrometer. (Ex/Em = 450/570). Values represent the means of three experiments. Means ± standard deviation; *p < 0.05, **p < 0.01, and ***p < 0.001 compared to untreated control group. ^#^ *p* < 0.05 and ^###^ *p* < 0.001 between the two groups.

Furthermore, chemotherapy increases the consumption of glutathione and sulfhydryl in cells, followed by an increase in ROS, which eventually leads to DNA damage (41). To determine whether DD induces ROS accumulation, a DCFDA staining assay kit was used. As shown in **Figures 5A, B**, the amount of intracellular ROS was significantly increased compared with that in the untreated control group in AML cells. To determine the role of ROS in DD-induced apoptosis, ROS was measured in AML cells with and without NAC pretreatment. NAC is an antioxidant and a safe and inexpensive drug that induces glutathione production and inhibits the depletion of MMP (ΔΨ) as an ROS scavenger (42). As shown in **Figures 5C, D**, ROS accumulation was effectively attenuated by NAC pretreatment compared with that in the untreated group in U937 and THP-1 cells. Consistently, the reduced cell viability caused by DD was significantly recovered by NAC pretreatment in both cells (**Figures 5E, F**). These findings showed that the DD-induced apoptosis in AML cells may depend on the regulation of ROS accumulation.

### DD Regulated the Expression Level of miR-216b Along and Inhibited p-c-Jun in AML Cells

**Figure 6:**
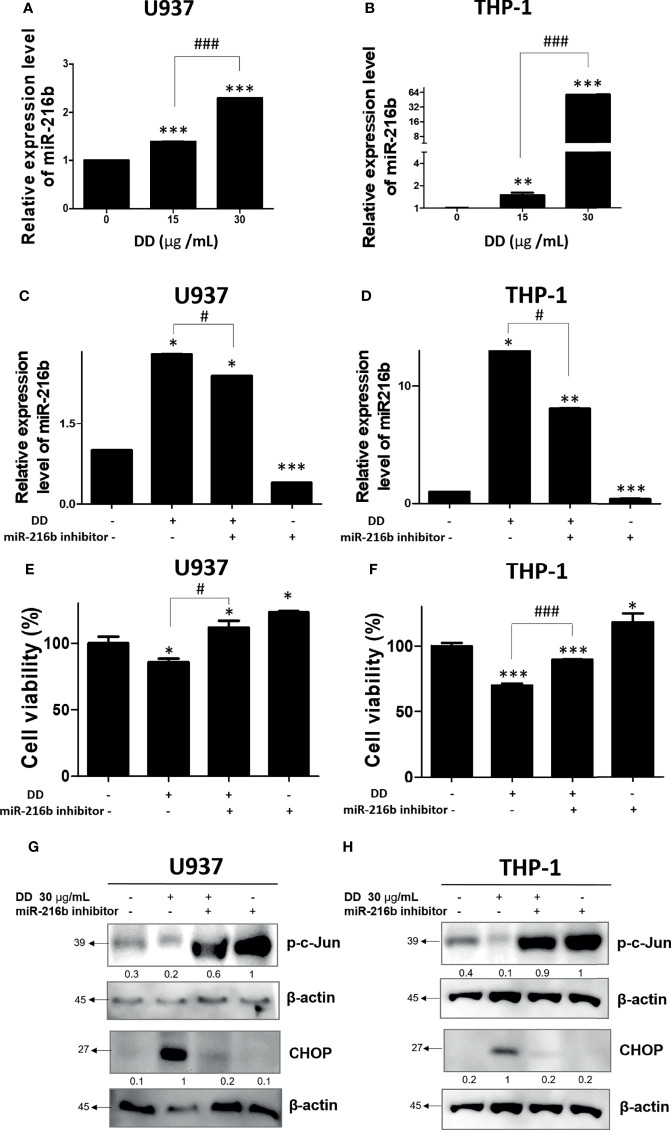
Anticancer effect of *Daemonorops draco* Blume (DD) and reactivation of c-Jun and CCAAT/enhancer-binding protein-mediated apoptosis in acute myeloid leukemia cells. DD elevated the expression of miR-216b in **(A)** U937 and **(B)** THP-1 cells. AML cells were transfected with miR-216b inhibitor. **(C–H)** A total of 1 × 10^5^ cells/ml were seeded into 6-well plates and allowed to reach approximately 50% density of transfection. The cells were transfected with miR-216b inhibitor for 48 h and exposed to the indicated doses of DD (i.e., 15 and 30 μg/ml) for 24 h. Following transfection for 48 h, miRNA was isolated and adopted to quantitative analysis of miRNA expression level or cell viability **(E, F)**, and Western blotting **(G, H)** was performed. Values represent the means of three experiments. Means ± standard deviation; *p < 0.05, **p < 0.01, and ***p < 0.001 compared with untreated control group. ^#^ *p* < 0.05, and ^###^ *p* < 0.001 between the two groups.

Several studies have identified c-Jun as a target protein of miR-216b, which was effective in alleviating cancer-related symptoms (26). Since DD significantly reduced c-Jun, its upstream miRNA, miR-216b, was measured. To measure the expression of miR-216b, qRT-PCR was performed in U936 and THP-1 cell lines. The treatment of DD significantly upregulated the expression of miR216b in a concentration-dependent manner (**Figures 6A, B**). To measure the role of miR-216b in DD-induced apoptosis, qRT-PCR and cell viability assay were performed. MiR-216b inhibitor transfection reversed the increased miR-216b by DD treatment (**Figures 6C, D**). Consistently, reduced cell viability by DD treatment was increased by miR-216 inhibitor transfection (**Figures 6E, F**). These results indicate that miR-216b is involved in the anticancer effects of DD. Additionally, to examine the involvement of miR-216b in the anticancer effect of DD and apoptosis, we performed qRT-PCR of miR-216b, together with Western blotting of p-c-Jun in AML cells, and we observed that miR-216b level was highly increased in THP-1 and moderately in U937 cells (**Figures 6A–D**). Transfection of miR-216b inhibitor in the presence of DD significantly upregulated p-c-Jun level and reduced CHOP, an ER stress-related apoptosis marker, compared to the DD-only treated cells (**Figures 6G, H**). Collectively, these results document that miR-216b-mediated c-Jun and CHOP are closely related to DD-induced apoptosis of AML cells.

## Discussion

AML is one of the most aggressive types of cancer with a low treatment success rate (43, 44). The survival rate of AML is estimated to be less than 10% when a relapse occurs (43, 45). Symptoms and prognosis of AML are associated with numerous gene mutations, which leads to the difficulty of making clear diagnosis and treatment decision. The mutation in hematopoietic stem cell that has the multipotent ability of self-renewal could be related to clonal expansion, making it the distinct characteristic responsible for the variability of AML (46). In a recent cohort study, 86% of patients showed two or more gene mutations, and co-mutated gene increased the mortality of AML (47). Induction therapy is the main treatment for patients with AML, using chemotactic agents, such as anthracyclines and cytarabine (48). Consolidation therapy is used in AML relapse or minimal residual leukemia, in which chemotherapy and hematopoietic stem cell transplantation are used alone or in combination. Recently, new treatment strategies, such as *FLT3* inhibitors, *IDH* inhibitors, nuclear exporter inhibitors, and immune therapies, are introduced to regulate genetic expression and immunological responses in AML (48). However, complete remission of chemotherapy in older patients is relatively low, while specific treatment methods for relapsed/refractory AML have yet to be identified (49). Furthermore, short-term and long-term side effects of chemotherapy were identified, accompanied by significant impacts on quality of life in patients with AML (50). Diverse variations in AML and the limitations of existing anticancer drugs suggest the need for research on alternative treatments for AML.

Meanwhile, the efficacy of DD in AML has not been explored, and the underlying mechanisms of DD were identified in U937 and THP-1 cells, including apoptosis, ROS, and miRNA regulation. U937 is a pro-monocytic, human myeloid leukemia cell line, which is commonly used to elucidate mechanisms of monocyte and macrophage differentiation. THP-1 is a human monocytic leukemia cell line, characterized by the expression of Fc and C3b receptors, with the lack of surface immunoglobulins (51). The classification of membrane receptors, such as IgG or C3b, is thought to be consistent with the AML model because they are detected in the blast cells of patients with AML. Blast cells are classified into myeloblasts, myelomonocytes, and monocytes (52).

Recently, clinical case studies have reported that the apoptosis pathway involving caspase 3 and cleaved PARP are major mediators that enhance chemotherapy effectiveness (53, 54). Moreover, cleaved caspase 3 triggers various pathways involved in apoptosis signaling (53). The mitochondrion is a sensor of apoptosis-promoted caspase activation in response to the apoptotic signaling pathway caused by DNA damage or various cellular stresses (55). Notably, the reduction in MMP (ΔΨ) is characterized by inevitable apoptosis resulting in the cleaved form of executioner caspase 3, causing the proteolysis of PARP (56). Here, the ER stress-related factor CHOP induces the activation of caspase 3 due to DNA damage caused by drug treatment, leading to apoptosis (56, 57). CHOP induced by numerous cellular stresses is a pro-apoptotic factor that promotes the activation of apoptotic genes and the hyper-oxidation of the ER lumen (58). Meanwhile, ATF4 plays a dual role in maintaining protein homeostasis while inducing apoptosis and cell cycle arrest. ATF4 is related to the reduction of stress in cancer cells due to lipid accumulation and malnutrition, as well as angiogenesis and metastasis, and conversely, when the situation changes, cancer cells are vulnerable to apoptosis through chemotherapy (59). Notably, ATF4 and CHOP prefer binding to similar motifs (GCATCAT/G) that share target gene sets (26). The forced expression of ATF4 and CHOP induced ATP depletion and oxidative stress protein synthesis that could result in cell death (52). Conversely, c-Jun N-terminal kinase (JNK), referred to as a serine/threonine (Ser/Thr) protein kinase, is included in the mitogen-activated protein kinase family. JNK mediates various cellular responses, such as proliferation, differentiation, survival, migration, invasion, and apoptosis, and stimulates inflammation, fibrosis, cancer progression, and metabolic diseases (60–62).

Thus, to determine the anticancer effect of DD on AML, in this study, the underlying apoptotic signaling of DD was studied in connection with the regulation of c-Jun or ER stress-mediated apoptosis signaling. Here, the viability of AML cells treated with DD was inhibited in a dose-dependent manner, indicating the anticancer effect of DD on AML cells (**Figure 2**). Consistently, DD altered MMP (ΔΨ) and increased the expression of cleaved PARP, cleaved caspase 3, p-ATF4, and CHOP due to the activation of the apoptotic pathway in a dose-dependent manner, implying the potent involvement of ER stress-related pathway and mitochondrial-mediated caspase activation signaling in the anticancer effect of DD (**Figure 3**).

Several studies have reported that excessive ROS can cause DNA damage, such as DNA double-strand break or DNA protein cross-linking break generation, illustrating their genotoxic nature (63–66). p-H2A.X is a marker of DNA damage due to DNA double-strand break (67, 68). The underlying anticancer effect of DD was associated with DNA damage followed by apoptosis induction at the living. Consistently, DD significantly increased p-H2A.X due to DNA damage, which was confirmed using DNA-binding polar fluorescent probe through confocal microscopy. The red fluorescent probe could not penetrate live cell membranes and selectively binds to the DNA of dead cells (69), indicating increased DNA damage due to DD treatment (**Figure 4**).

Furthermore, to confirm whether the DD-induced apoptosis signaling is regulated by ROS, NAC pretreatment was performed. Importantly, NAC pretreatment alone or NAC with DD treatment increased cell viability compared with untreated controls, indicating that ROS accumulation plays a critical role in DD-induced activation of the apoptotic pathway in AML cells (**Figure 5**). Accumulating evidence showed that ER stress-related and mitochondria-mediated pathways are closely involved in ROS accmulation, thereby exerting potential anticancer effects of DD. The Human Genome Project has provided the genetic blueprint of humans and helps to uncover the genetic causes of human diseases including cancer. As human genome sequences have begun to be solved using algorithms, cancer progression has been found to be caused by miRNA, which controls tumor suppressor genes and oncogenes. Therefore, numerous studies on miRNA related to human diseases and cancer have been conducted (70). Of note, the importance of natural products in regulating miRNA has only recently begun to focus on determining therapeutic targets for cancer (71). Consistently, several studies have reported that herbal extracts are extensively related to modulating miRNA in association with the inhibition of epithelial–mesenchymal transition, chemoresistance, and metastasis (72, 73). Interestingly, miRNA is easy to acquire from patient blood or tissue samples and is used as a diagnostic and prognostic marker as it provides crucial information concerning gene expression profiling (74), suggesting that cancer-specific characterization of AML contributes to the advantage of targeting of miRNA-based therapy. Therefore, to assess whether DD has a miRNA-based therapeutic effect on AML, the underlying anticancer effect of DD was investigated in association with the miR-216b-mediated pathway. Here, DD significantly induced the expression of miR-216b in both U937 and THP-1 cells, revealing that DD is a regulator of miR-216b. Notably, it has been recently identified that plants significantly inhibit miRNA, which is required for controlling processes by the introduction of a sponge RNA involved in fine-tuning targeting miRNA (75, 76). MiR-216b directly inhibited c-Jun in response to ER stress, which led to CHOP-dependent apoptosis (26). Additionally, c-Jun is involved in cell survival in various cancers by the dysregulation of the PI3K/AKT axis, including NSCLC (77) and gastric cancer (61).

**Figure 7:**
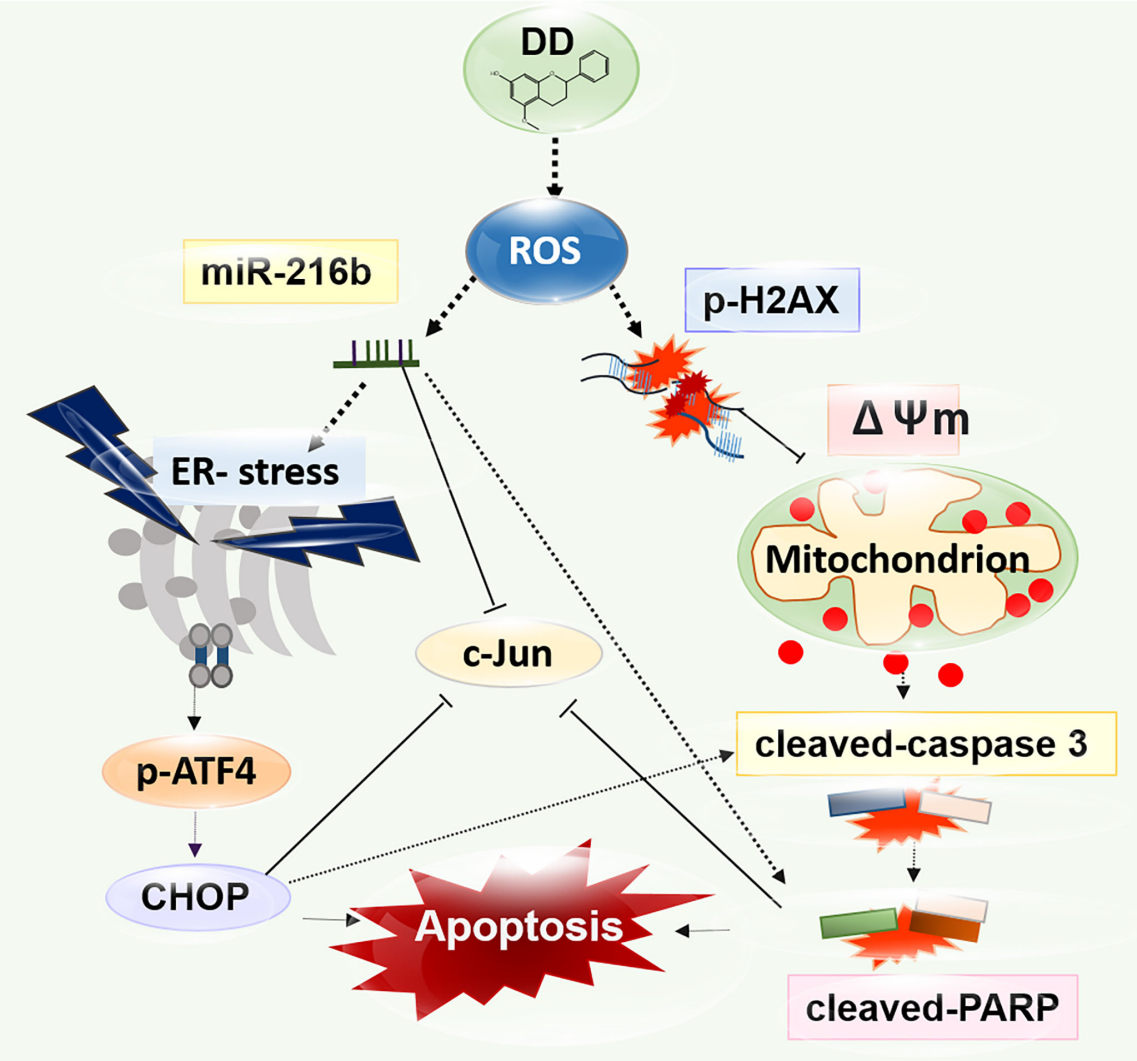
A diagram of the anticancer effect of *Daemonorops draco* Blume (DD) research strategy. DD significantly promoted mitochondria-mediated caspase activation and endoplasmic reticulum stress-related apoptosis pathway involved in reactive oxygen species generation, leading to apoptosis due to DNA damage in AML cells. DD treatment significantly showed anticancer effects on AML cells by significantly inhibiting c-Jun, leading to apoptosis *via* the regulation of miR-216b.

Interestingly, the excessive depletion of miR-216b and the activation of c-Jun by miR-216b inhibitor were observed compared with those in groups treated with DD alone, indicating that DD induces apoptosis in sensitized AML cells *via* miR-216b-dependent signaling (**Figure 6**). Additionally, the biological effects of natural products have been studied for decades, and recent analysis methods related to HPLC-MS have enabled gathering scientific data on effective compounds (78). Furthermore, it is well documented that effective compounds of natural products, such as alkaloids, phenolics, and carotenoids, have apoptotic effects on AML (79). *Salvia miltiorrhiza*, another traditional herbal medicine, classified similarly with DD in terms of blood circulatory effects, induces apoptosis and necrosis in a ROS-independent and caspase-independent manner in acute lymphoblastic leukemia cells (80). Additionally, *Spatholobi caulis*, an effective Chinese medicine for relieving blood stasis, was proved to exert caspase-dependent apoptotic activity on U937, a human monocyte leukemia cell line (81). In previous studies, compared with the efficacy of natural products targeting a single mechanism for leukemia caused by complex mutations, DD significantly activated mitochondria-mediated caspase activation, ER stress-related regulation of ROS, c-Jun, and miR-216b, indicating the multiple anticancer mechanisms of DD (**Figure 7**). However, the prepared DD solution is highly concentrated and bioavailability and inability could be the issues to achieve the therapeutic dose *in vivo*. Further investigation is needed for future studies about the anticancer effect of DD. The antitumor properties of DD should be examined further in in-depth studies on the specific therapeutic application methods of DD in treating AML. One of the constituents of DD, dracorhodin has been reported to have an anticancer effect in melanoma (82), esophageal squamous cell carcinoma (83), lung cancer (84), breast cancer (85), etc. Other compounds from DD including abietic acid (86) and nordracorubin (87) showed anticancer activities. DD could be a potent candidate for *in vivo* and clinical studies. This study is limited in that the effects of DD on AML are confined to *in vitro* studies. In future studies, the practical effects of DD should be explored through *in vivo* experiments. The determination of the specific interrelationships by which ROS lowered the viability of AML cells and regulated ER stress should be further addressed. Furthermore, to confirm the efficacy of DD in patients with AML, further studies are required for the identification of proper dosage of DD through animal experiments; moreover, studies on the subtypes of various AML cell lines and miRNA genes should be conducted.

## Conclusions

DD has a significant cytotoxic effect on AML cells. Notably, DD treatment efficiently induced ROS-mediated ER-associated degradation, including CHOP and p-ATF4 along with cleaved caspase 3 and cleaved PARP, and attenuated c-Jun, activating p-γH2A.X. Moreover, DD treatment regulated miR-216b-dependent ER stress-related apoptosis in AML cells. Overall, this study opens up the possibility of therapeutic application of DD against AML, involving upregulation of miR-216b.

## Data Availability Statement

The original contributions presented in the study are included in the article/supplementary material. Further inquiries can be directed to the corresponding authors.

## Author Contributions

Conceptualization and writing—original draft preparation: MP and HJ. Formal analysis: SJ, SP, and KK. Data curation: SJ and SP. Writing—review and editing: MR, S-HK, and BK. Visualization: MP, SJ, and KK. Supervision: S-HK, WK, and BK. Project administration: BK. Funding acquisition: BK. All authors contributed to the article and approved the submitted version.

## Funding

This research was supported by Basic Science Research Program through the National Research Foundation of Korea (NRF) funded by the Ministry of Education (NRF-2020R1I1A2066868), the National Research Foundation of Korea (NRF) grant funded by the Korea government (MSIT) (No. 2020R1A5A2019413), a grant of the Korea Health Technology R&D Project through the Korea Health Industry Development Institute (KHIDI), funded by the Ministry of Health & Welfare, South Korea (grant number: HF20C0116), and a grant of the Korea Health Technology R&D Project through the Korea Health Industry Development Institute (KHIDI), funded by the Ministry of Health & Welfare, South Korea (grant number: HF20C0038).

## Conflict of Interest

The authors declare that the research was conducted in the absence of any commercial or financial relationships that could be construed as a potential conflict of interest.

## Publisher’s Note

All claims expressed in this article are solely those of the authors and do not necessarily represent those of their affiliated organizations, or those of the publisher, the editors and the reviewers. Any product that may be evaluated in this article, or claim that may be made by its manufacturer, is not guaranteed or endorsed by the publisher.
